# Lipid Nanoparticle-Mediated RNAi Against GIPC1 Overcomes Chemoresistance in Pancreatic Ductal Adenocarcinoma

**DOI:** 10.3390/pharmaceutics17101334

**Published:** 2025-10-15

**Authors:** Vijay Sagar Madamsetty, Hari Krishnareddy Rachamala, Shamit Kumar Dutta, Enfeng Wang, Krishnendu Pal, Debabrata Mukhopadhyay

**Affiliations:** 1 Department of Biochemistry and Molecular Biology, Mayo Clinic College of Medicine and Sciences, Jacksonville, FL 32224, USArachamala.hari@mayo.edu (H.K.R.); pal.krishnendu@mayo.edu (K.P.); 2 Department of Physiology and Biomedical Engineering, Mayo Clinic College of Medicine and Sciences, Jacksonville, FL 32224, USA

**Keywords:** pancreatic ductal adenocarcinoma (PDAC), GIPC1, RNA interference (RNAi), siRNA delivery, lipid nanoparticles (LNPs)

## Abstract

**Background/Objectives:** Pancreatic ductal adenocarcinoma (PDAC) remains one of the most lethal malignancies, characterized by aggressive tumor biology, poor vascularization, dense stromal barriers, and profound resistance to chemotherapy. GAIP-interacting protein C-terminus 1 (GIPC1), a PDZ-domain-containing adaptor protein, is highly overexpressed in PDAC and plays a critical role in tumor progression and chemoresistance. This study aimed to develop and evaluate a novel tumor-targeted liposomal siRNA delivery system (LGIPCsi) to silence GIPC1 and enhance the therapeutic efficacy of gemcitabine (GEM) in PDAC; **Methods:** LGIPCsi nanoparticles were synthesized and optimized for physicochemical stability, siRNA complexation efficiency, and tumor-targeting capability. Their therapeutic efficacy was assessed using in vitro pancreatic cancer cell models and in vivo orthotopic and patient-derived xenograft (PDX) models of PDAC. Biodistribution, tumor uptake, and antitumor efficacy were evaluated following systemic administration. Combination studies were performed to assess the synergistic effects of LGIPCsi and GEM; **Results:** GIPC1 silencing significantly sensitized pancreatic cancer cells to GEM, resulting in enhanced inhibition of tumor cell proliferation in vitro. In vivo, systemic administration of LGIPCsi achieved efficient intratumoral delivery of siGIPC1, leading to marked tumor growth suppression. Combination therapy with GEM and LGIPCsi produced synergistic antitumor effects, with substantial tumor regression compared to monotherapy groups. Importantly, no significant systemic toxicity was observed in treated animals; **Conclusions:** This study identifies GIPC1 as a promising therapeutic target in PDAC and demonstrates that tumor-targeted siRNA nanomedicine can effectively overcome chemoresistance when combined with standard chemotherapy. The LGIPCsi platform offers a rational and translational strategy to enhance treatment efficacy in PDAC through targeted RNAi-based combination therapy.

## 1. Introduction

PDAC presents as an aggressive malignancy with restricted therapeutic options and a dismal 5-year survival rate of approximately 13.3% [1,2]. Early diagnosis is challenging, often resulting in advanced disease at presentation, and chemoresistance frequently emerges in pancreatic cancer cells, complicating treatment regimens [3]. Additionally, the dense fibrous stroma surrounding pancreatic tumors acts as a barrier to drug delivery, exacerbating treatment resistance [4]. The molecular and cellular heterogeneity of PDAC further complicates the development of targeted therapies [5,6]. Patients with PDAC commonly experience compromised overall health, limiting their tolerance for intensive treatments like surgery or chemotherapy [3]. Current chemotherapeutic agents and combination regimens, including GEM, Nab-Paclitaxel, Fluorouracil (5-FU), Oxaliplatin, Irinotecan, and FOLFIRINOX, exhibit modest efficacy against the aggressive and chemo-resistant PDAC [7]. Novel therapeutic strategies are urgently needed to improve patient outcomes and enhance the quality of life in PDAC treatment, specifically given the current young age at presentation and the significant increase in pancreatic cancer.

The GIPC PDZ domain has emerged as a critical therapeutic target due to its pivotal role in stabilizing Insulin-like growth factor 1 receptor (IGF-1R), Dopamine receptor D2 (DRD2), Transforming growth factor β receptor 3 (TGFβR3), LDL receptor-related protein 1 (LRP1), Neuropilin 1 (NRP1), Glucose transporter type 1 (GLUT1), Syndecan 4 (SDC4), Semaphorin 4C (SEMA4C), and Integrin α5 (ITGA5), which are involved in tumorigenesis pathways [8,9]. Given its central role in PDAC pathophysiology and its extensive involvement in multiple oncogenic pathways, targeting GIPC1 presents a promising strategy to overcome therapeutic resistance and enhance treatment efficacy. In this regard, we developed and reported the cell-permeable linear lipopeptide CR1023 (N-myristoyl-PSQSSSEA), which mimics the C-terminal sequence of GAIP [10] and significantly inhibited PDAC cell proliferation in vitro and suppressed tumor growth in vivo, demonstrating the potential of targeting GIPC1 for cancer therapy. Further, we designed and developed a series of side chain-modified derivatives of CR1023 featuring halogenated aromatic side chains that showed higher activity than CR1023, downregulating EGFR/IGF-1R expression in pancreatic and breast cancer models [11]. However, peptide-based inhibitors face several translational challenges, including rapid enzymatic degradation and renal clearance limiting their half-life, potential immune responses and off-target interactions, and lower binding affinity requiring higher doses and chemical modifications [12]. In addition, developing a small-molecule inhibitor for targets containing a PDZ motif, such as GIPC1, has proven to be unsuccessful thus far.

A major obstacle in cancer therapy is the creation of delivery vehicles capable of transporting drugs or genetic material specifically to tumor tissues with high efficiency. Over the past several decades, research on tumor-targeted delivery strategies has expanded considerably, leading to the development of a variety of nanoscale carriers, typically around 100 nm in size, to enhance drug distribution and intratumoral accumulation [13,14]. Despite the encouraging outcomes from many preclinical studies, translation into the clinic has been modest: only a limited number of passively targeted nanoformulations have gained regulatory approval, and none of the actively targeted formulations have advanced successfully beyond clinical trials [15]. A notable example of clinical progress is the approval of liposomal irinotecan in combination regimens for metastatic pancreatic cancer. At the same time, nucleic acid–based therapeutics have emerged as promising anticancer agents, though their widespread application remains hampered by delivery barriers. To address this, multiple approaches have been developed to facilitate efficient nucleic acid transport into tumors. The approval of the first RNAi-based drug for the treatment of polyneuropathy marked a new era for the drug delivery field, demonstrating the therapeutic feasibility of nucleic acid medicines. Moving forward, clinical translation will require delivery platforms that rely on biocompatible, clinically validated materials and allow reproducible, controllable synthesis processes [7,16].

The purpose of this study was to develop and evaluate a novel tumor-targeted liposomal delivery system for RNAi against GIPC1 in PDAC. Given the critical role of GIPC1 in stabilizing multiple oncogenic receptors and driving chemoresistance, this study aimed to determine whether targeted silencing of GIPC1 using siRNA could sensitize PDAC cells and tumors to GEM. By optimizing an LGIPCsi for stability, siRNA encapsulation, and selective tumor uptake, the research sought to overcome delivery challenges and assess the therapeutic efficacy of GIPC1 silencing alone or in combination with GEM in orthotopic and patient-derived xenograft models. Ultimately, the goal was to establish GIPC1 as a promising therapeutic target and demonstrate the translational potential of RNAi nanomedicine as a rational combinatorial strategy for PDAC treatment.

## 2. Materials and Methods

### 2.1. Reagents and Materials

Dulbecco’s Modified Eagle Medium (DMEM; Gibco, Waltham, MA, USA), RPMI-1640 medium (Gibco, Waltham, MA, USA), and DMEM/F-12 medium (Gibco) supplemented with fetal bovine serum (FBS; tetracycline-free; Gibco, Waltham, MA, USA), Antibiotic–Antimycotic (Gibco), and Plasmocin (InvivoGen, San Diego, CA, USA) were used for cell culture. Antibodies included anti-β-actin and HRP-conjugated secondary antibodies (Santa Cruz Biotechnology, Dallas, TX, USA) and anti-GIPC antibody (Thermo Fisher Scientific, Waltham, MA, USA). Tet-On–inducible GIPC1 shRNA plasmids were purchased from Dharmacon, Lafayette, CO, USA. Puromycin (2 μg/mL; Sigma-Aldrich, St. Louis, MO, USA) and doxycycline (0.4 μg/mL for in vitro and 0.5 mg/mL for in vivo; Sigma-Aldrich, St. Louis, MO, USA) were used for selection and induction, respectively. Cell viability was assessed using the CellTiter-Glo^®^ 2.0 Assay (Promega, Madison, WI, USA). For protein extraction and quantification, NP-40 lysis buffer, protease inhibitor cocktail, and Halt phosphatase inhibitor cocktail (Thermo Fisher Scientific, Waltham, MA, USA) were employed, and protein concentrations were determined using the Pierce™ BCA Protein Assay Kit (Thermo Fisher, Waltham, MA, USA, Cat. #23225). For Western blotting, 6× Laemmli SDS sample buffer (Bio-Rad, Hercules, CA, USA), PVDF membranes (Millipore, Burlington, MA, USA), and SuperSignal™ West Pico Chemiluminescent Substrate (Thermo Scientific, Waltham, MA, USA) were used. Liposomal formulations were prepared with KRC18 (custom lipid), DOPC, DOPE, and DSPE-PEG2000-Amine (Avanti Polar Lipids, Alabaster, AL, USA), cholesterol (Sigma-Aldrich, St. Louis, MO, USA), TTLP (custom tumor-targeting ligand peptide), and Rhodamine-PE (Avanti Polar Lipids, Alabaster, AL, USA). GIPC1 siRNA was custom synthesized (Dharmacon, Lafayette, CO, USA). Agarose gels and ethidium bromide (Bio-Rad, Hercules, CA, USA) were used for gel retardation assays. Hydrodynamic diameter and zeta potential were measured using a Zetasizer (Malvern Instruments, Malvern, UK, Zetasizer Software version v7.13), and Milli-Q water (Millipore, Burlington, MA, USA) was used for formulation stability studies. For in vivo imaging, sodium D-Luciferin (Gold Biotechnology, St. Louis, MO, USA, Cat. #LUCK-100) was administered, and animals were imaged using the IVIS Imaging System (Waltham, MA, USA) with Living Image Software (IVIS Living Image Software version v4.7). For RT-qPCR, TRIzol™ Universal Reagent (Thermo Fisher Scientific, Waltham, MA, USA), iScript™ cDNA Synthesis Kit (Bio-Rad, Hercules, CA, USA, Gol Cat. #1708890), and SYBR^®^ Green Premix Ex Taq™ II (Tli RNaseH Plus; Takara, San Jose, CA, USA, Cat. #RR820A) were used, and amplification was performed on an Applied Biosystems QuantStudio™ 5 Real-Time PCR System (Applied Biosystems, Foster City, CA, USA).

### 2.2. Cell Culture

Human pancreatic cancer cell lines PANC-1 and AsPC-1 were obtained from the American Type Culture Collection (ATCC), whereas patient-derived xenograft (PDX) cell lines, including 6741, 4636, 4535, and 4911, were established from primary tumor specimens collected from pancreatic cancer patients at the Mayo Clinic. PANC-1 cells were maintained in Dulbecco’s Modified Eagle Medium (DMEM, Gibco, Waltham, MA, USA), and AsPC-1 cells were maintained in RPMI-1640 medium. Primary pancreatic cancer cells (6741, 4636, 4535, and 4911) were maintained in DMEM/F-12. All cell culture media were supplemented with 10% fetal bovine serum (FBS); tetracycline-free FBS (10%) was specifically used to prepare the medium for maintaining GIPC-1–inducible cell lines. In addition, media were supplemented with 1% anti-anti (penicillin–streptomycin, Gibco, Waltham, MA, USA) to prevent bacterial contamination and 0.02% plasmocin (InvivoGen, San Diego, CA, USA) to prevent mycoplasma contamination.

### 2.3. Animals

Six- to eight-week-old *SCID* mice were obtained from the in-house breeding colony and maintained in the institutional animal facility. All animal experiments were performed with the highest standards of care in compliance with the guidelines of the Association for Assessment and Accreditation of Laboratory Animal Care (AAALAC) and under protocols approved (Protocol A00004002-18) by the Mayo Clinic Institutional Animal Care and Use Committee (IACUC), further ensuring the ethical conduct of our research.

### 2.4. Antibodies

Antibodies for β-actin and horseradish peroxidase-conjugated secondary antibodies were purchased from Santa Cruz Biotechnology. Anti-GIPC antibody was purchased from Thermo Fisher, Waltham, MA, USA.

### 2.5. shRNA Transfection

The plasmids for Tet-on-inducible GIPC1 shRNA were purchased from Dharmacon, Lafayette, CO, USA. Lentivirus stocks for GIPC1 shRNA were prepared and infected into the target cells following standard procedures. For antibiotic selection, 2 μg/mL of puromycin was added to the medium. For the Tet-On-inducible GIPC shRNA, 0.4 μg/mL of doxycycline was used to induce shRNA expression in the stably infected cells.

### 2.6. In Vitro Cytotoxicity Assay

Approximately 5 × 10^3^ cells were seeded in 384-well plates. After 18–24 h, cells were treated with increasing concentrations of GEM and incubated for a further 72 h. Cell viabilities were assayed using the CellTiter-Glo^®^ 2.0 Assay (Promega, Madison, WI, USA) as per the manufacturer’s protocol. Briefly, 24 μL of reagent was added to each well. The plates were incubated for 10 min at RT, and luminescence signals were measured using Spectramax i3x (Molecular Devices, LLC (San Jose, CA, USA), version 7.x. Percentage viability is calculated as follows: Viability (%) = 100 × (A_Treated_ − A_Blank_)/(A_Untreated_ − A_Blank_).

### 2.7. Western Blot

For in vitro studies, cells were washed three times with ice-cold PBS and lysed using NP-40 lysis buffer supplemented with 1% protease inhibitor cocktail and 1% Halt phosphatase inhibitor cocktail (Pierce, Rockford, IL, USA ). The lysates were centrifuged at 13,000 rpm for 20 min at 4 °C, and the supernatants were collected. Protein concentrations were quantified using the BCA assay (Pierce BCA Protein Assay Kit, Waltham, MA, USA).

For in vivo samples, proteins were denatured by adding 6× Laemmli SDS sample buffer and heating at 95 °C for 5 min. Equal amounts of protein were loaded into each lane for SDS-PAGE, followed by wet transfer onto PVDF membranes. Membranes were blocked in TBS-T buffer (50 mmol/L Tris-HCl, pH 7.4; 150 mmol/L NaCl; 0.05% Tween-20) containing 5% nonfat milk or BSA. Membranes were then incubated overnight at 4 °C with the primary antibody diluted in TBS-T containing 5% nonfat milk or BSA, followed by incubation for 1 h at room temperature with horseradish peroxidase (HRP)-conjugated secondary antibody (Santa Cruz Biotechnology, Dallas, TX, USA). Protein bands were visualized using the SuperSignal West Pico Chemiluminescent Substrate (Thermo Scientific, Waltham, MA, USA).

### 2.8. In Vivo Biodistribution of FAM-siRNA and NIR Dye-Loaded LGIPCsi

Six- to eight-week-old male SCID mice were obtained from in-house breeding and housed in the institutional animal facilities. All animal work was performed under protocols approved by the Mayo Clinic Institutional Animal Care and Use Committee. To establish orthotopic pancreatic tumors, approximately 1 × 10^6^ luciferase-labeled PANC-1 cells resuspended in 100 μL of PBS were injected into the pancreas of each mouse. Tumors were allowed to grow for 4 weeks without treatment. LGIPCsi loaded with IR-780 dye and complexed with FAM-siRNA was administered via the intravenous route. Mice were imaged using the IVIS imager 24 and 48 h after administration.

### 2.9. In Vivo Tumor Growth Inhibition Study with GIPC1 shRNA Plasmids and GEM

A single mouse trial (SMT) was used to assess the in vivo tumor inhibition study in combination with GIPC-1 knockdown plus GEM treatment. This strategy is currently being used in ‘Avatar’ models for the development of personalized therapy for various diseases. These ‘Avatar’ models are developed by implanting patient tumor samples in mice for subsequent drug efficacy studies. Each tumor-bearing mouse is treated with a different therapeutic regimen to identify the most effective regimen, thereby reducing the cost and toxicity associated with non-targeted therapy. We used the same strategy in our SMT. All experiments were accomplished according to the permitted guidelines. For the pancreatic cancer orthotopic model, 6-8-week-old female SCID mice were obtained from in-house breeding. Approximately 1 × 10^6^ luciferase-labeled Tet-inducible GIPC-1 shRNA vector-transfected AsPC-1 or PANC-1 cells, resuspended in 100 μL of PBS, were injected into the pancreas of each mouse. After three days of cell inoculation, GIPC-1 knockdown was induced by adding doxycycline (0.5 mg/mL) in drinking water for the remainder of this study. After substantial tumor growth was confirmed by bioluminescence imaging, mice were treated with GEM (10 mg/kg) twice a week for four weeks. After completion of the experiment, all tumor-bearing mice were euthanized with CO_2_; tumors were harvested, and tumor volumes and weights were measured. We also performed a validation study to confirm the results obtained from the SMT in AsPC-1 tumor-bearing mice with 5 mice per group.

### 2.10. Preparation of Liposomes

LGIPCsi lipid nanoparticles were prepared using the thin film hydration method. LGIPCsi consisted of KRC18, DOPC, DOPE, cholesterol, DSPE-(PEG-2000)-amine, and TTLP in a weight ratio of 0.5:3.98:0.35:0.484:0.27:0.22. For fluorescent labeling, 50 µg of Rh-PE was added. Ingredients were dissolved in chloroform, and the solvents were evaporated with nitrogen gas and dried under a high vacuum for 4 h. The film was hydrated for 12 h with water or siRNA solution. For LGIPCsi, the film was hydrated with GIPC-siRNA pre-condensed with KRC18. The suspensions were vortexed, sonicated, and subjected to 8 freeze/thaw cycles. Sequential extrusions through 200 nm and 100 nm pore-size membranes yielded 100–150 nm unilamellar liposomes.

### 2.11. Gel Binding Assay

The siRNA binding ability of LGIPCsi was assessed by gel retardation assay on a 1.2% agarose gel pre-stained with ethidium bromide. The LGIPCsi complex was incubated with 2% SDS at room temperature for 20–25 min. Then, 2 µL of 6X loading buffer was added, and the solution was loaded into the wells. Samples were electrophoresed at 80 V for 45 min, and siRNA bands were visualized using a Gel Documentation System (Bio-Rad, Hercules, CA, USA).

### 2.12. Zeta Potential and Hydrodynamic Diameter Measurements

HDD and zeta potentials (1:100 dilution) were measured in triplicate by using a Zetasizer (Malvern, UK). For stability studies, liposomes were incubated in autoclaved Milli-Q water at 4 °C for up to 30 days, with size measurements taken at various intervals.

### 2.13. In Vivo Tumor Growth Inhibition Study with GIPC-1 siRNA and GEM

To harness the therapeutic efficacy of GIPC-1 knockdown in vivo, we developed a novel liposomal siRNA delivery system with a tumor-targeting ligand (LGIPCsi). Luciferase-labeled PANC-1 cells were used to develop orthotopic PDAC xenografts in 6-8-week-old female SCID mice following a similar procedure described above. After 4 weeks, tumor growth was confirmed by bioluminescence imaging, and tumor-bearing mice were divided into five groups (*n* = 5). The mice were i.p. Administered with: LNPs, LGIPCsi (0.5 mg/kg) complexed with LGIPCsi, GEM (10 mg/kg), and a combination of both twice a week for three weeks. The untreated group was used as a control. The LGIPCsi and GEM treatments were performed two days apart. After completing the experiment, all tumor-bearing mice were euthanized with CO_2_; tumors were harvested for morphologic analysis and immunostaining.

### 2.14. Bioluminescent Imaging

Tumor growth of orthotopic tumors was analyzed by bioluminescent imaging. Sodium-D-Luciferin (Gold Biotechnology, St. Louis, MO, USA) was injected intraperitoneally at 100 mg/kg in PBS 5 min before imaging. Mice were anesthetized using isoflurane and imaged with the IVIS system, Xenogen, Waltham, MA, USA. Signals were displayed as photons/second/cm^2^/steradian and were measured using the Living Image software (IVIS Living Image Software, v4.7).

### 2.15. Reverse Transcription Quantitative Real-Time PCR (RT-qPCR)

Total RNA was isolated from tumor samples subjected to different treatments using the TRIzol™ Universal Reagent (Thermo Fisher Scientific, Waltham, MA, USA) according to the manufacturer’s protocol. Complementary DNA (cDNA) was synthesized from purified RNA using the iScript™ cDNA Synthesis Kit (Bio-Rad, Hercules, CA, USA RR037). Quantitative real-time PCR was performed with SYBR^®^ Green Premix Ex Taq™ II (Tli RNaseH Plus, RR820A; Takara, San Jose, CA, USA) following the supplier’s instructions. Gene-specific primers used for amplification are provided below. PCR reactions were conducted on an Applied Biosystems QuantStudio™ 5 Real-Time PCR System (Applied Biosystems, Foster City, CA, USA). Relative mRNA expression was normalized to β-Actin as the internal control and calculated using the 2^−ΔΔCT^ method. All assays were performed in triplicate to ensure reproducibility.
**Gene****Species****Forward****Reverse**GIPC-1HumanCCTGATGGTGGACCAGAGGTGGTACACCCCTCCTAATGCCIGFR betaHumanTTTTGACTCCGCGTTTCTGCCAAAGAAAGGGGGCAAAGCC

### 2.16. Statistical Analyses

Data were analyzed using Microsoft Excel (v2312, Microsoft Office 365) and GraphPad Prism (v9.4.0). One-way ANOVA or unpaired two-tailed *t*-tests were used to assess differences between treatment groups, as appropriate. For tumor growth curves, endpoint or same-day tumor volumes were compared using unpaired two-tailed *t*-tests. Statistical significance was defined as *p* < 0.05 (*), *p* < 0.01 (**), *p* < 0.001 (***), and *p* < 0.0001 (****). Error bars represent standard deviation (SD).

## 3. Results

### 3.1. GIPC-Depletion In Vitro Sensitizes Pancreatic Cancer Cells Towards GEM

**Figure 1:**
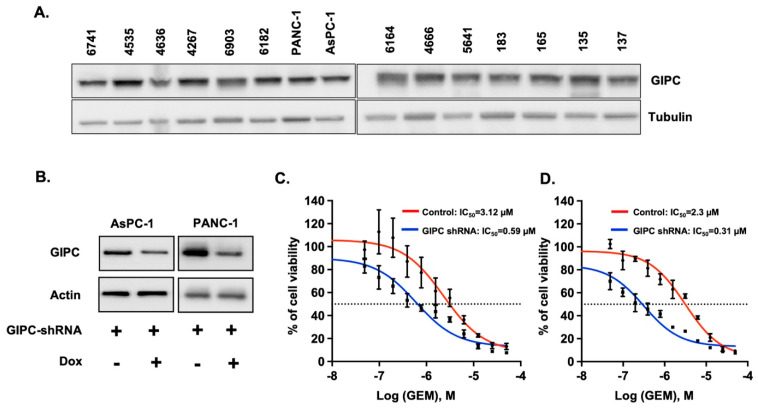
**In vitro tumorigenic properties of GIPC in PDAC development.** (**A**) Western blot analysis showing endogenous expression levels of GIPC in patient-derived xenograft (PDX), PANC-1, and AsPC-1 pancreatic cancer cell lines. (**B**) Western blot analysis confirming GIPC knockdown following treatment with doxycycline (400 ng/mL, 72 h) in Tet-inducible shRNA AsPC-1 and PANC-1 cells. Actin was used as a loading control. (**C**) AsPC-1 and (**D**) PANC-1 cells with doxycycline-pretreated GIPC knockdown showed increased sensitivity to GEM compared with doxycycline-untreated controls, as assessed by CellTiter Glo assay.

Before experiments to evaluate the effect of GIPC1 depletion in sensitizing pancreatic cancer cells towards chemotherapeutic drugs, we analyzed the GIPC1 expression levels in AsPC-1, PANC-1, and some PDX cell lines. As shown in Figure 1A, GIPC1 expression could be seen in all the cell lines, corroborating the previous notion that GIPC1 is an essential survival gene in pancreatic cancer. Next, we intended to see whether GIPC1 depletion in pancreatic cancer cells can increase their sensitivity towards chemotherapeutic drugs such as GEM. Towards this end, we utilized lentiviral-based GIPC1 shRNA plasmids to perform GIPC1 knockdown in four cell lines. Unfortunately, we failed to generate stable clones of GIPC-depleted PDAC cell lines since the growth of the cells was not adequate to generate stable cell lines. Therefore, we had to use the Tet-on inducible smart-GIPC1 shRNA system. We analyzed three different clones of Tet-on inducible GIPC1 shRNA for their GIPC1 knockdown efficacy in AsPC-1 GFP-Luciferase cells and selected the best clone demonstrating the highest knockdown after induction with 400 ng/ml doxycycline for 72 h for further in vitro drug sensitivity experiments (Figure 1B). For these experiments, we added increasing concentrations of GEM to doxycycline-pretreated or untreated AsPC-1 and PANC-1 cells across a concentration range from 0.5 µM to 50 µM. The MTS assay was performed after 72 h. GIPC-depleted cells exhibited a steeper growth inhibition curve compared to the control cells, suggesting that GIPC-depletion sensitizes pancreatic cancer cells towards GEM in vitro (Figure 1C,D).

### 3.2. GIPC-Depletion Sensitizes Orthotopic Pancreatic Cancer Xenografts Towards GEM In Vivo

**Figure 2:**
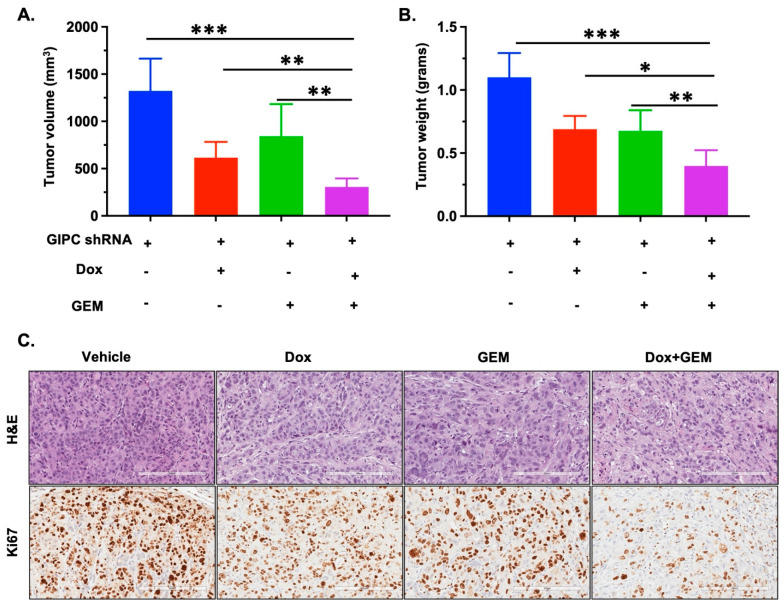
In vivo tumorigenic properties of GIPC in pancreatic cancer growth in a single mouse experiment and validation in pancreatic cancer. GIPC knockdown was achieved by treating with 0.5 mg/mL of doxycycline in water after three days of cell implantation and continued throughout this study. In an in vivo study, endpoint results clearly suggest that GIPC knockdown increases GEM efficacy in mice treated 2×/3 wk with 10 mg/kg of GEM in AsPC-1: (**A**) tumor volume and (**B**) tumor weight. The GIPC shRNA group without Dox or GEM is shown in blue, the GIPC shRNA group with Dox is shown in red, the GIPC shRNA group with GEM is shown in green, and the GIPC shRNA group with Dox + GEM is shown in purple. Error bars represent standard deviation (SD). Statistical significance was defined as *p* < 0.05 (∗), *p* < 0.01 (∗∗), and *p* < 0.001 (∗∗∗). (**C**) Immunohistochemistry of tumor tissues for H&E and Ki67 (Scale bar 200 μm) for different treatment groups.

To further validate our hypothesis in vivo, we performed a single mouse trial using two pancreatic cancer cell lines, namely AsPC-1 and PANC-1. We observed that GIPC-depleted tumors were significantly smaller in both PANC-1 (Figure S1A,B) and AsPC-1 (Figure S1C,D) orthotopic xenografts. Also, GIPC-depleted tumors exhibited a significantly higher response to GEM treatment for both cell lines. To validate the results obtained from the single mouse trail, we repeated the same experiment in AsPC-1 xenografts with five mice per treatment group as usual. We observed more or less similar results in that experiment (Figure 2A,B). Histological evaluation of H&E- and Ki67-stained tumor sections revealed that GIPC1-depleted tumors displayed pronounced morphological alterations accompanied by a marked downregulation of proliferative markers (Figure 2C). Taken together, these results suggest that GIPC knockdown sensitizes pancreatic cancer xenografts towards GEM in vivo. Additionally, the single mouse trial’s usefulness in identifying the best treatment strategy for combating cancer was also substantiated.

### 3.3. Targeted Liposomal Formulation of GIPC1 siRNA Validated for Encapsulation, Cellular Uptake, and Functional Activity in PDAC

Based on our findings and corroborating evidence from previous studies, GIPC1 has emerged as a promising therapeutic target in PDAC. Nonetheless, earlier attempts to strongly modulate this pathway did not yield sufficient therapeutic efficacy [10,11,17]. We therefore hypothesized that selective inhibition of GIPC1-mediated signaling could represent a more effective strategy for PDAC treatment. To overcome the lack of efficient small-molecule inhibitors against GIPC1, our group developed a tumor-targeting lipopeptide (TTL) capable of directing therapeutics to tumor tissue [7,18]. Using this lipopeptide, we engineered a novel tumor-targeted liposomal formulation encapsulating GIPC1-specific siRNA (LGIPCsi).

**Figure 3:**
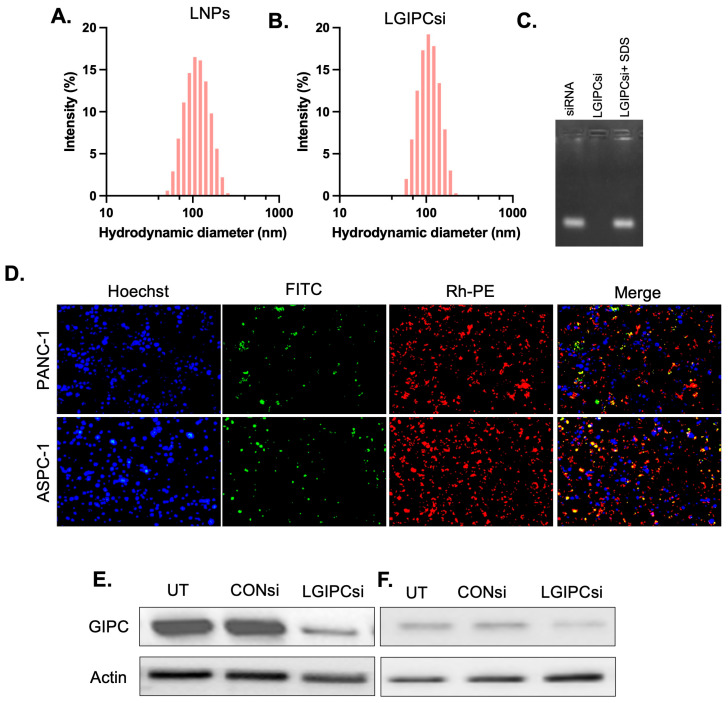
Hydrodynamic sizes of empty lipid nanoparticles (LNPs) (**A**) and GIPC1 siRNA-loaded LNPs (LGIPCsi) (**B**). All the measurements were performed in deionized water at 25 °C. (**C**) Agarose gel electrophoresis: Relative electrophoretic mobilities of free siRNA (lane 1), LGIPCsi (lane 2), and LGIPCsi treated with 0.1% SDS for 30 min at 37 °C (lane 3). In each case, 0.5 µg of siRNA was used. (**D**) In vitro cellular uptake of FITC-siRNA complexed and Rhodamine-PE-labeled liposomes in PDAC cell lines. PANC1 cells (top panel) and AsPC-1 cells (lower panel) were treated with FITC-siRNA complexed and Rhodamine-PE-labeled liposomes for four hours. The nuclei of the cells were counterstained with Hoechst for the last 30 min, and images were captured using an EVO fluorescence microscope under blue, red, and green channels. Scale bar length = 200 μm. (**E**,**F**) Knockdown of GIPC gene expression in PDAC cells using LGIPCsi liposomes. PANC1 (**E**) and AsPC-1 (**F**) cells were transfected with lipoplexes with control siRNA (CONsi), GIPCsiRNA (last lane), and untreated samples (first lane) in each cell line. GIPC protein expression was determined in cell lysates by Western blotting.

Using a combination of ionic lipids, phospholipids, helper lipids, PEGylated lipids, TTL, and GIPC1-specific siRNA, we developed the LGIPCsi formulation. The physicochemical properties of the liposomes were characterized using dynamic light scattering (DLS), which revealed their hydrodynamic diameter and surface potential (Figure 3A,B; Supplementary Table S1). We evaluated the stability of the LGIPCi formulation in four different media: Milli-Q water, DMEM buffer, DMEM supplemented with 10% FBS, and DMEM supplemented with 55% FBS, for up to seven days. In Milli-Q water (103 nm) and DMEM buffer (106 nm), the hydrodynamic diameters remained stable without significant changes over the seven-day period. In contrast, in DMEM containing 10% FBS (176 nm), the particle size remained stable only up to five days, after which a marked increase in size was observed, rendering the formulation unstable and unmeasurable. Similarly, in DMEM containing 55% FBS (234 nm), stability was maintained for only two days before the particle size increased drastically, preventing further measurements (Figure S2). Compared with water and DMEM buffer, the presence of serum proteins in FBS appeared to promote particle aggregation, likely due to the interaction of negatively charged serum proteins with the positively charged lipid nanoparticles, resulting in increased particle size and reduced stability (Supplementary Table S1). To verify siRNA encapsulation, we performed agarose gel electrophoresis. Naked GIPC1 siRNA (lane I) exhibited a distinct band, whereas no free siRNA was detected when encapsulated within the liposomes (lane II), confirming near-complete encapsulation efficiency. Upon liposomal disruption with 0.1% SDS, the encapsulated siRNA was released and clearly detected (lane III), further validating the efficient entrapment of GIPCSi within the liposomal system (Figure 3C). Fluorescence microscopy demonstrated efficient cellular uptake of FITC-siRNA–loaded, Rhodamine-PE–labeled liposomes in both PANC1 and AsPC-1 pancreatic cancer cell lines. After 4 h of incubation, strong green (FITC-siRNA) and red (Rhodamine-PE) signals were observed in the cytoplasm, with substantial overlap around the perinuclear region, confirming successful delivery of siRNA via the liposomal carrier. Hoechst counterstaining of nuclei further confirmed intracellular localization of the nanocomplexes (Figure 3D).

To evaluate the functional effect of delivery, GIPC1 gene knockdown was assessed following liposomal siRNA transfection. Both PANC1 and AsPC-1 cells treated with GIPC1-siRNA liposomes showed a marked reduction in GIPC1 expression compared with untreated controls and cells transfected with control siRNA (Figure 3E). These findings confirm that the formulated liposomes not only facilitate efficient siRNA uptake but also induce robust gene silencing in PDAC cells.

### 3.4. LGIPCsi Demonstrates Tumor-Selective Uptake in Orthotopic Pancreatic Cancer Xenografts

The results from the above experiments prompted us to assess the therapeutic potential of GIPC1 depletion in vivo. Towards this end, we developed a novel tumor-targeted liposomal formulation (LGIPCsi) for siRNA delivery in vivo. To evaluate the tumor-targeting efficacy of the LGIPCsi formulation, we performed an in vivo tumor uptake study in PANC-1 orthotopic tumor-bearing mice. FAM-siRNA complexed with tumor-targeted (LGIPCsi) liposomal formulations was injected intraperitoneally in orthotopic PANC-1 tumor-bearing mice. In vivo tumor uptake was monitored by IVIS imaging after 24 h and 48 h. There is no autofluorescence interfering with the signal intensity from mice fur in this region as well. As shown in Figure S3, our novel tumor-targeting liposomal formulation LGIPCsi clearly showed significant tumor uptake compared to the control. However, we did not observe a clear signal of FAM-siRNA due to strong autofluorescence in the green region of the spectrum.

### 3.5. Combined GIPC-1 Silencing and GEM Treatment Synergistically Suppressed Tumor Growth and Proliferation

The therapeutic efficacy of GIPC1 silencing in combination with GEM was evaluated in an orthotopic AsPC-1 pancreatic xenograft mouse model. Mice were randomized into five groups (n = 5 per group): untreated control, LNPs, GEM (10 mg/kg), GIPC1 siRNA (0.5 mg/kg), and the combination of GEM with GIPC1 siRNA. Treatments were administered twice weekly for three weeks.

**Figure 4:**
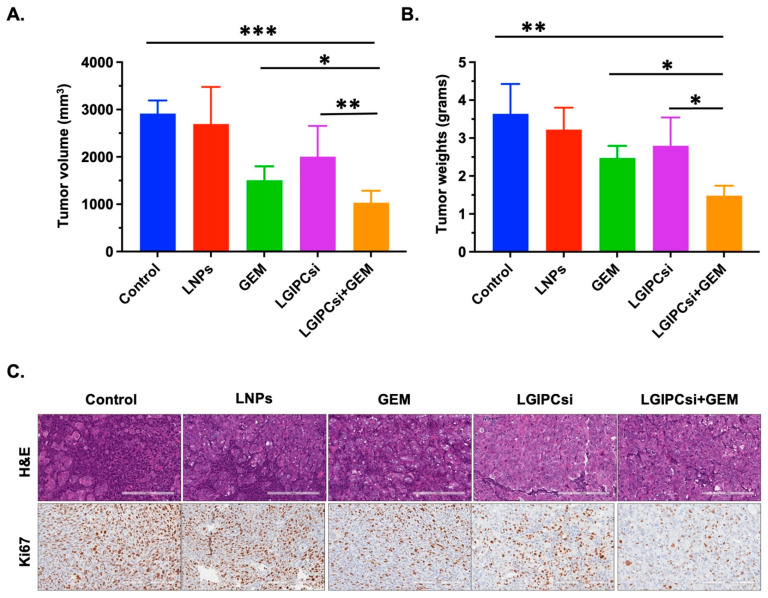
Anti-tumor effects of the combination of downregulating of GIPC-1 and GEM treatments in a pancreatic xenograft mouse model: Mice bearing AsPC-1 orthotopic pancreatic tumors were treated with five different combinations (n = 5): empty liposome (LNPs), GEM (10 mg/kg), GIPC-1 siRNA (0.5 mg/kg), both GEMs (10 mg/kg), and GIPC-1 siRNA (0.5 mg/kg) for over 2x/3 weeks. The untreated group was used as a control. (**A**) Tumor volume, (**B**) Tumor weight. The antitumor efficacy of GEM combined with GIPC-1 siRNA-treated mice significantly inhibited tumor growth compared with only GEM-treated or only GIPC siRNA-treated or control mice. Error bars represent standard deviation (SD). Statistical significance was defined as *p* < 0.05 (∗), *p* < 0.01 (∗∗), and *p* < 0.001 (∗∗∗). (**C**) Immunohistochemistry of tumor tissues for H&E and Ki67 (Scale bar 200 μm) for different treatment groups.

As shown in Figure 4A,B, combination therapy with GEM and GIPC1 siRNA produced a significant reduction in both tumor volume and tumor weight compared with monotherapies or control groups. Neither empty liposomes nor single-agent treatments achieved comparable tumor suppression, underscoring the synergistic benefit of the combination regimen. Histological analyses further supported these findings. H&E staining revealed reduced tumor cellularity in the combination group, while Ki67 immunostaining demonstrated a marked decrease in proliferative index compared with GEM alone, GIPC1 siRNA alone, or controls (Figure 4C). These results confirm that concurrent downregulation of GIPC1 enhances GEM efficacy, leading to robust inhibition of tumor growth in PDAC xenografts.

**Figure 5:**
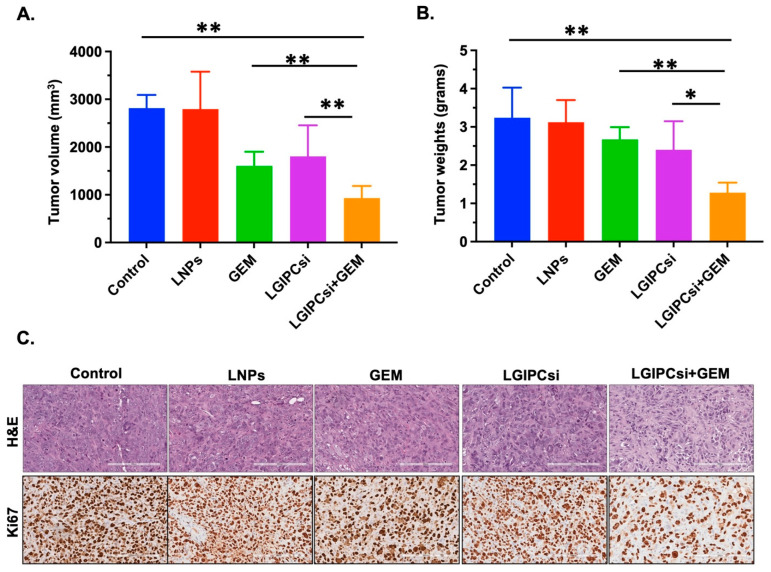
Anti-tumor effects of the combination of downregulating of GIPC-1 and GEM treatments in a pancreatic xenograft mouse model: Mice bearing PANC-1 orthotopic pancreatic tumors were treated with five different combinations (n = 5): empty liposome (LNPs), GEM (10 mg/kg), GIPC-1 siRNA (0.5 mg/kg), both GEMs (10 mg/kg), and GIPC-1 siRNA (0.5 mg/kg) for over 2×/3 weeks. The untreated group was used as a control. (**A**) Tumor volume, (**B**) Tumor weight. The antitumor efficacy of GEM combined with GIPC-1 siRNA-treated mice significantly inhibited tumor growth compared with only GEM-treated or only GIPC siRNA-treated or control mice. Error bars represent standard deviation (SD). Statistical significance was defined as *p* < 0.05 (∗), and *p* < 0.01 (∗∗). (**C**) Immunohistochemistry of tumor tissues for H&E and Ki67 (Scale bar 200 μm) for different treatment groups.

The impact of combined GIPC1 silencing and GEM treatment was further evaluated in an orthotopic PANC-1 pancreatic xenograft model. Mice were assigned to five groups (n = 5 each): untreated control, empty liposome (LNPs), GEM (10 mg/kg), GIPC1 siRNA (0.5 mg/kg), or the combination of GEM with GIPC1 siRNA, administered twice weekly for three weeks. As in Figure 5A,B, the combination treatment produced a marked reduction in both tumor volume and tumor weight compared with GEM alone, GIPC1 siRNA alone, or control groups. Neither empty liposomes nor monotherapies demonstrated comparable tumor inhibition. Histopathological analysis provided further confirmation. H&E staining revealed reduced tumor cellularity in the combination group, while Ki67 immunostaining demonstrated a substantial decrease in proliferative activity relative to single-agent or control groups (Figure 5C). Collectively, these findings show that concurrent GIPC1 knockdown significantly enhances the antitumor efficacy of GEM in PANC-1 xenografts.

**Figure 6:**
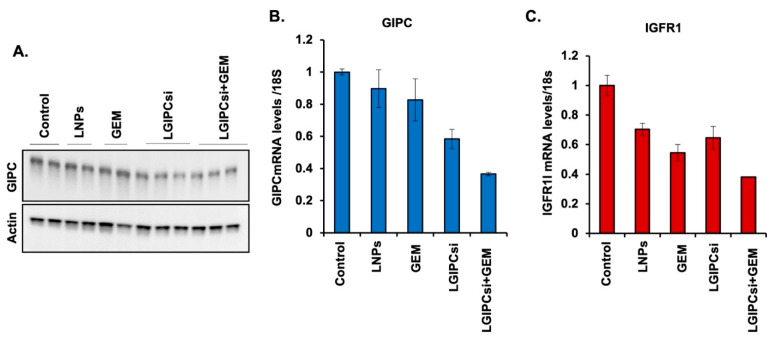
Western blot and RT-PCR analysis of the combination of downregulating of GIPC-1 and GEM treatments in a pancreatic xenograft mouse model: Mice bearing PANC-1 orthotopic pancreatic tumors were treated with five different combinations (n = 5): empty liposome (LNPs), GEM (10 mg/kg), GIPC-1 siRNA (0.5 mg/kg), both GEMs (10 mg/kg), and GIPC-1 siRNA (0.5 mg/kg) for over two times per week upto three weeks. The untreated group was used as a control. (**A**) Western blot showing GIPC kd in treatment. The antitumor efficacy of GEM combined with GIPC-1 siRNA-treated mice significantly inhibited tumor growth compared with only GEM-treated or only GIPC siRNA-treated or control mice. (**B**) GIPC and (**C**) IGFR1 mRNA levels of different treatment groups of tumor tissues.

Western blot analysis demonstrated efficient downregulation of GIPC-1 expression in tumors treated with GIPC-1 siRNA, confirming effective gene silencing in vivo (Figure 6A). RT-PCR analysis further supported these findings, showing marked reductions in GIPC-1 mRNA levels in siRNA-treated tumors compared with controls. Notably, the combination of GIPC-1 siRNA and GEM resulted in the most pronounced suppression of GIPC-1 expression relative to either monotherapy (Figure 6B). In addition, IGFR1 mRNA expression, a downstream signaling mediator of GIPC-1, was significantly decreased in tumors from the combination treatment group, suggesting effective disruption of GIPC-1–IGFR1–mediated oncogenic signaling (Figure 6C). These molecular changes correlated with the enhanced antitumor efficacy observed in the combination group compared with single-agent or control treatments.

### 3.6. Combination of GIPC-1 Silencing and GEM Exhibits Robust Antitumor Efficacy Across Multiple Pancreatic Xenograft Models

In the single-mouse orthotopic pancreatic xenograft experiments using 6741, 4636, 4535, and 4911 tumor models, combination treatment with GIPC-1 siRNA and GEM consistently demonstrated superior antitumor efficacy compared to individual treatments or controls (Figure S4). In the 6741 tumors, both tumor volume and tumor weight were markedly reduced in the combination group relative to the control, empty liposome, GEM alone, or GIPC-1 siRNA alone. Similar trends were observed in the 4636 tumors, where the combination treatment induced substantial inhibition of tumor growth, confirming that dual targeting of GIPC-1 and chemotherapy enhances therapeutic outcomes. In the 4535 and 4911 tumor models, combination-treated mice exhibited the most significant suppression of tumor progression, as reflected by reductions in both tumor burden and final tumor weights, whereas monotherapies produced only modest effects. Collectively, these findings across four independent pancreatic tumor models validate the robust and reproducible antitumor activity of combined GIPC-1 knockdown and GEM therapy, highlighting the synergistic potential of this strategy to overcome the limited efficacy of monotherapies.

## 4. Discussion

PDAC remains one of the most aggressive malignancies, with limited therapeutic options and dismal patient survival. Despite advances in surgical techniques and systemic therapies, durable responses are rare, and resistance to chemotherapy is nearly universal [7]. Thus, there is an urgent need for novel therapeutic strategies that can overcome drug resistance and improve patient outcomes. Building on our previous work demonstrating that GIPC1 is overexpressed in PDAC and associated with poor prognosis [10,11], the present study was designed to develop and evaluate a novel tumor-targeted liposomal delivery system (LGIPCsi) for RNA interference against GIPC1. Given the critical role of GIPC1 in stabilizing multiple oncogenic receptors and driving chemoresistance, we sought to determine whether targeted silencing of GIPC1 using siRNA could sensitize PDAC cells and tumors to gemcitabine (GEM). By optimizing the LGIPCsi formulation for stability, siRNA encapsulation, and selective tumor uptake, we aimed to overcome delivery challenges and assess the therapeutic efficacy of GIPC1 silencing alone or in combination with GEM in orthotopic and patient-derived xenograft models. Ultimately, this research establishes GIPC1 as a promising therapeutic target and highlights the translational potential of RNAi-based nanomedicine as a rational combinatorial strategy for PDAC treatment.

These findings underscore the therapeutic potential of targeting GIPC1 in pancreatic cancer. GIPC1 is abundantly expressed in PDAC and functions as a critical signaling hub by interacting with the PDZ domain of diverse cell-surface receptors and signaling proteins [19]. Through these interactions, GIPC1 orchestrates pathways essential for cell survival, migration, proliferation, and metabolic adaptability [20]. Stable knockdown or pharmacologic interference with the GIPC1 PDZ domain has previously been shown to inhibit tumor growth, highlighting its importance in PDAC progression [11]. In line with this, our results demonstrate that siRNA-mediated knockdown of GIPC1 not only impairs tumor cell proliferation in vitro but also enhances the sensitivity of PDAC cells and xenografts to GEM.

At present, no selective small-molecule inhibitors of GIPC1 are available. This prompted us to employ RNA interference (RNAi) as a targeted approach to suppress GIPC1 expression. The therapeutic relevance of siRNA has been validated by the recent approval of the first RNAi-based drug, supporting its clinical feasibility [21]. However, siRNAs face several challenges, including poor stability, limited cellular uptake, rapid clearance, and insufficient tissue penetration [22]. To overcome these limitations, we developed a liposomal nanoparticle system capable of encapsulating and delivering GIPC1-specific siRNA to pancreatic tumors. Our results demonstrate efficient encapsulation, robust cellular uptake, and effective gene silencing both in vitro and in vivo.

GEM remains the backbone of PDAC treatment; however, its clinical benefit is severely limited by innate and acquired resistance. While combination regimens such as FOLFIRINOX have improved outcomes in selected patients, these approaches are associated with high toxicity and reduced quality of life [2]. Our study provides evidence that GIPC1 silencing significantly enhances the antitumor efficacy of GEM in orthotopic PDAC models. Tumors treated with the combination therapy exhibited profound reductions in volume and proliferative index compared with monotherapy groups. Mechanistically, this synergistic effect appears to involve disruption of GIPC1-mediated stabilization of pro-survival receptors, including IGF-1R and GLUT1, leading to impaired PI3K/AKT signaling and metabolic support. Collectively, these results establish GIPC1 silencing as a rational strategy to overcome GEM resistance and improve therapeutic efficacy.

Beyond PDAC, GIPC1 has been implicated in several other malignancies. It regulates migration and invasion in breast cancer through MyoGEF–Cdc42–MMP9 signaling, facilitates ECM remodeling via interactions with neuropilin-1 and α5β1 integrin, and sustains receptor trafficking in glioma and melanoma. In glioblastoma and ovarian cancer, GIPC1 promotes angiogenesis by amplifying VEGF signaling [8]. These diverse oncogenic roles highlight the broad relevance of GIPC1 as a therapeutic target and suggest that strategies developed for PDAC may have applicability across multiple tumor types.

Despite its importance, direct pharmacological targeting of PDZ domains remains a major challenge [23]. Their shallow binding pockets, structural conservation, and promiscuous ligand interactions hinder the development of high-affinity, selective inhibitors [24]. While peptide-based inhibitors of GIPC1 have shown promise in preclinical studies, their instability, poor bioavailability, and immunogenicity limit translational potential [10,11]. Small-molecule PDZ inhibitors have thus far been largely unsuccessful [25]. Emerging advances in artificial intelligence (AI)-driven drug discovery may help overcome these barriers by enabling the rational design of drug-like scaffolds with improved pharmacological properties [26]. AI-based pipelines have already yielded several first-in-class molecules with high Phase I success rates, suggesting that the development of selective PDZ-targeting agents, including those against GIPC1, may soon be achievable.

The translational relevance of our study is reinforced by the growing clinical validation of RNAi therapeutics and the regulatory success of lipid-based nanomedicines [27]. Delivering GIPC1 siRNA via tumor-targeted liposomes provides a feasible approach for clinical application. Moreover, our findings suggest that single-mouse xenograft trials can serve as an efficient screening tool to identify effective therapeutic combinations before proceeding to large-scale preclinical studies, thereby accelerating translational progress.

Nevertheless, limitations remain. While our orthotopic and patient-derived xenograft models recapitulate many aspects of PDAC biology, they do not fully capture the complexity of the stromal and immunosuppressive tumor microenvironment that characterizes human disease. Additionally, durable GIPC1 suppression may require optimization of siRNA formulations or integration with emerging genome-editing strategies. Future studies exploring combinatorial approaches such as GIPC1 silencing with immune checkpoint blockade or AI-designed small-molecule inhibitors may further enhance therapeutic outcomes.

In summary, improving the overall survival of patients with PDAC will require identifying therapeutic targets and strategies to target them and means to deliver therapies to the tumors. In the present study, we developed a novel tumor-targeted liposomal formulation for delivering RNAi to the tumor site. Studied extensive characterization of these liposomal formulations. Bioactivities of the formulations were thoroughly evaluated in vitro and in vivo by protein expression, bio-distribution study, and tumor growth inhibition study. Intravenous administration of the liposomal formulation complexed with GIPC1 siRNA inhibited tumor growth and significantly increased the GEM sensitivity in orthotopic pancreatic xenografts compared to that for the untreated control group. The presently described tumor-targeted liposomal formulation may find future use in combating pancreatic cancer through simultaneous in vivo delivery of a small molecule-based cytotoxic drug and anti-cancer siRNA.

## Data Availability

Data is contained within the article or Supplementary Materials.

## Supplementary Materials

The following supporting information can be downloaded at: https://www.mdpi.com/article/10.3390/pharmaceutics17101334/s1, Figure S1: In-vivo tumorigenic properties of GIPC in pancreatic cancer growth in single mice experiment and validation in pancreatic cancer.; Table S1: Hydrodynamic diameter (nm), surface charge (mV), and polydispersity index (PDI) of the lipid nanoparticles measured in four different mediums: Figure S2. Stability of LGIPCsi formulations in four different mediums: Figure S3: In vivo selective tumor uptake of targeted liposomal formulations. Figure S4: Anti-tumor effects of combination of downregulating of GIPC-1 and Gemcitabine treatments in in single mice experiment and validation in pancreatic xenograft mouse model.

## References

[1] NIH Cancer Stat Facts: Pancreatic Cancer. https://seer.cancer.gov/statfacts/html/pancreas.html.

[2] Beutel A.K., Halbrook C.J. (2023). Barriers and opportunities for gemcitabine in pancreatic cancer therapy. Am. J. Physiol. Cell Physiol..

[3] Halbrook C.J., Lyssiotis C.A., Pasca di Magliano M., Maitra A. (2023). Pancreatic cancer: Advances and challenges. Cell.

[4] He Y., Zhang W., Xiao Q., Fan L., Huang D., Chen W., He W. (2022). Liposomes and liposome-like nanoparticles: From anti-fungal infection to the COVID-19 pandemic treatment. Asian J. Pharm. Sci..

[5] Seidu T.A., Kutoka P.T., Asante D.O., Farooq M.A., Alolga R.N., Bo W. (2022). Functionalization of Nanoparticulate Drug Delivery Systems and Its Influence in Cancer Therapy. Pharmaceutics.

[6] Cheng Q., Wei T., Farbiak L., Johnson L.T., Dilliard S.A., Siegwart D.J. (2020). Selective organ targeting (SORT) nanoparticles for tissue-specific mRNA delivery and CRISPR-Cas gene editing. Nat. Nanotechnol..

[7] Rachamala H.K., Nakka N.M.R., Angom R.S., Bhattacharya S., Pal K., Mukhopadhyay D. (2025). Dual Targeting of Syndecan-1 and Glucose Transporter-1 With a Novel Lipid-Based Delivery System Enhances Therapeutic Efficacy and Overcomes Chemoresistance in Pancreatic Ductal Adenocarcinoma. Gastroenterology.

[8] Ahmed T., Mythreye K., Lee N.Y. (2021). Strength and duration of GIPC-dependent signaling networks as determinants in cancer. Neoplasia.

[9] Chen C., Chen C., Li Y., Gu R., Yan X. (2023). Characterization of lipid-based nanomedicines at the single-particle level. Fundam. Res..

[10] Patra C.R., Rupasinghe C.N., Dutta S.K., Bhattacharya S., Wang E., Spaller M.R., Mukhopadhyay D. (2012). Chemically modified peptides targeting the PDZ domain of GIPC as a therapeutic approach for cancer. ACS Chem. Biol..

[11] Muders M.H., Vohra P.K., Dutta S.K., Wang E., Ikeda Y., Wang L., Udugamasooriya D.G., Memic A., Rupasinghe C.N., Baretton G.B. (2009). Targeting GIPC/synectin in pancreatic cancer inhibits tumor growth. Clin. Cancer Res..

[12] Boafo G.F., Magar K.T., Ekpo M.D., Qian W., Tan S., Chen C. (2022). The Role of Cryoprotective Agents in Liposome Stabilization and Preservation. Int. J. Mol. Sci..

[13] Imtiaz S., Ferdous U.T., Nizela A., Hasan A., Shakoor A., Zia A.W., Uddin S. (2025). Mechanistic study of cancer drug delivery: Current techniques, limitations, and future prospects. Eur. J. Med. Chem..

[14] Liu B., Zhou H., Tan L., Siu K.T.H., Guan X.-Y. (2024). Exploring treatment options in cancer: Tumor treatment strategies. Signal Transduct. Target. Ther..

[15] Wang B., Hu S., Teng Y., Chen J., Wang H., Xu Y., Wang K., Xu J., Cheng Y., Gao X. (2024). Current advance of nanotechnology in diagnosis and treatment for malignant tumors. Signal Transduct. Target. Ther..

[16] Paunovska K., Loughrey D., Dahlman J.E. (2022). Drug delivery systems for RNA therapeutics. Nat. Rev. Genet..

[17] Muders M.H., Dutta S.K., Wang L., Lau J.S., Bhattacharya R., Smyrk T.C., Chari S.T., Datta K., Mukhopadhyay D. (2006). Expression and regulatory role of GAIP-interacting protein GIPC in pancreatic adenocarcinoma. Cancer Res..

[18] Rachamala H.K., Madamsetty V.S., Angom R.S., Nakka N.M., Dutta S.K., Wang E., Mukhopadhyay D., Pal K. (2024). Targeting mTOR and survivin concurrently potentiates radiation therapy in renal cell carcinoma by suppressing DNA damage repair and amplifying mitotic catastrophe. J. Exp. Clin. Cancer Res..

[19] Borchardt H., Schulz A., Datta K., Muders M.H., Aigner A. (2019). Silencing of Neuropilins and GIPC1 in pancreatic ductal adenocarcinoma exerts multiple cellular and molecular antitumor effects. Sci. Rep..

[20] Li T., Zhong W., Yang L., Zhao Z., Wang L.I., Liu C., Li W., Lv H., Wang S., Yan J. (2023). GIPC1 promotes tumor growth and migration in gastric cancer via activating PDGFR/PI3K/AKT signaling. Oncol. Res..

[21] Li X.-J., Nie P., Herdewijn P., Sun J.-G. (2023). Unlocking the synthetic approaches and clinical application of approved small-molecule drugs for gastrointestinal cancer treatment: A comprehensive exploration. Eur. J. Med. Chem..

[22] Lu D., Sun Y., Luan Y., He W. (2024). Rational design of siRNA-based delivery systems for effective treatment of brain diseases. Pharm. Sci. Adv..

[23] Gutiérrez-González L.H., Rivas-Fuentes S., Guzmán-Beltrán S., Flores-Flores A., Rosas-García J., Santos-Mendoza T. (2021). Peptide Targeting of PDZ-Dependent Interactions as Pharmacological Intervention in Immune-Related Diseases. Molecules.

[24] Christensen N.R., Čalyševa J., Fernandes E.F.A., Lüchow S., Clemmensen L.S., Haugaard-Kedström L.M., Strømgaard K. (2019). PDZ Domains as Drug Targets. Adv. Ther..

[25] Kamdem N., Roske Y., Kovalskyy D., Platonov M.O., Balinskyi O., Kreuchwig A., Saupe J., Fang L., Diehl A., Schmieder P. (2021). Small-molecule inhibitors of the PDZ domain of Dishevelled proteins interrupt Wnt signalling. Magn. Reson..

[26] Khan M.K., Raza M., Shahbaz M., Hussain I., Khan M.F., Xie Z., Shah S.S.A., Tareen A.K., Bashir Z., Khan K. (2024). The recent advances in the approach of artificial intelligence (AI) towards drug discovery. Front. Chem..

[27] Saw P.E., Song E. (2024). Advancements in clinical RNA therapeutics: Present developments and prospective outlooks. Cell Rep. Med..

