# Supplementary material for: Lipid Nanoparticle-Mediated RNAi Against GIPC1 Overcomes Chemoresistance in Pancreatic Ductal Adenocarcinoma
Source: Pharmaceutics. 2025 Oct 15;17(10):1334. doi: 10.3390/pharmaceutics17101334 (PMC12567285; doi:10.3390/pharmaceutics17101334)
Supplement: Supplementary file 1 [file pharmaceutics-17-01334-s001.zip › pharmaceutics-3868224-supplementary.docx]

**Supplementary Materials**

**Lipid Nanoparticle-Mediated RNAi Against GIPC1 Overcomes Chemoresistance in Pancreatic Ductal Adenocarcinoma**

Vijay Sagar Madamsetty, Hari Krishnareddy Rachamala, Shamit Kumar Dutta, Enfeng Wang, Krishnendu Pal, Debabrata Mukhopadhyay

**B**

**A**


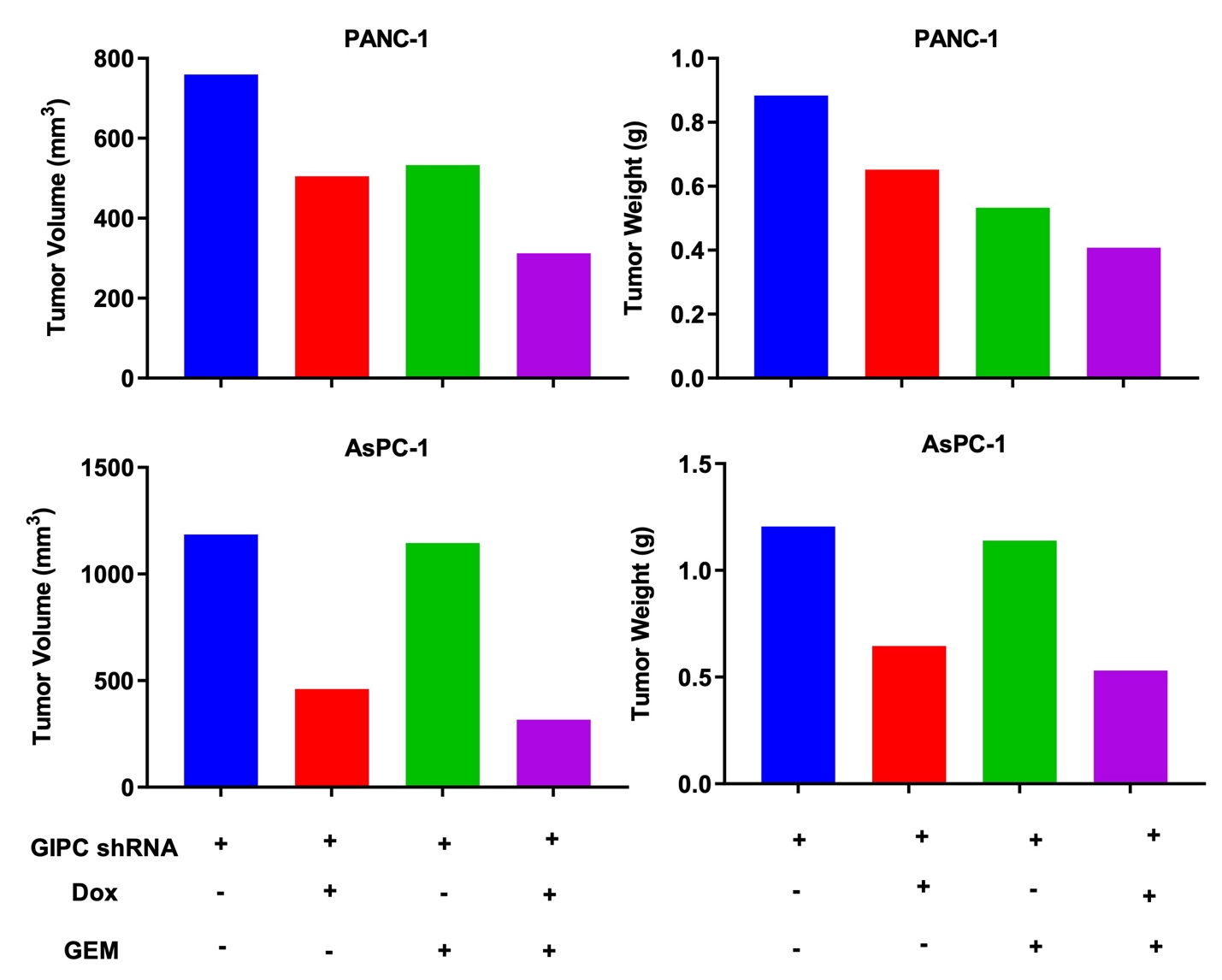


**D**

**C**

**Figure S1. In vivo tumorigenic role of GIPC in pancreatic cancer and its impact on gemcitabine efficacy:** The tumorigenic properties of GIPC were evaluated using single-mouse experiments in PANC-1 and AsPC-1 xenograft models. GIPC knockdown was induced by administering doxycycline (0.5 mg/mL) in drinking water starting three days post tumor cell implantation and continued throughout the study. Mice were treated with gemcitabine at 10 mg/kg, twice weekly for three weeks (2×/3 wk). Tumor growth inhibition was assessed by measuring (A) tumor volume and (B) tumor weight in PANC-1, and (C) tumor volume and (D) tumor weight in AsPC-1 xenografts. The treatment groups are represented as follows: GIPC shRNA without Dox or GEM (blue), GIPC shRNA with Dox (red), GIPC shRNA with GEM (green), and GIPC shRNA with Dox + GEM (purple). Endpoint analyses clearly indicate that GIPC knockdown enhances the therapeutic efficacy of gemcitabine in both PANC-1 and AsPC-1 models.

**Supplementary Table S1.** Hydrodynamic diameter (nm), surface charge (mV), and polydispersity index (PDI) of the lipid nanoparticles measured in four different media: Milli-Q water, DMEM buffer, DMEM supplemented with 10% FBS, and DMEM supplemented with 55% FBS.

**Figure S2.** Stability of LGIPCsi formulations in four different media, Milli-Q water, DMEM buffer, DMEM with 10% FBS, and DMEM with 55% FBS, monitored for up to seven days after preparation.


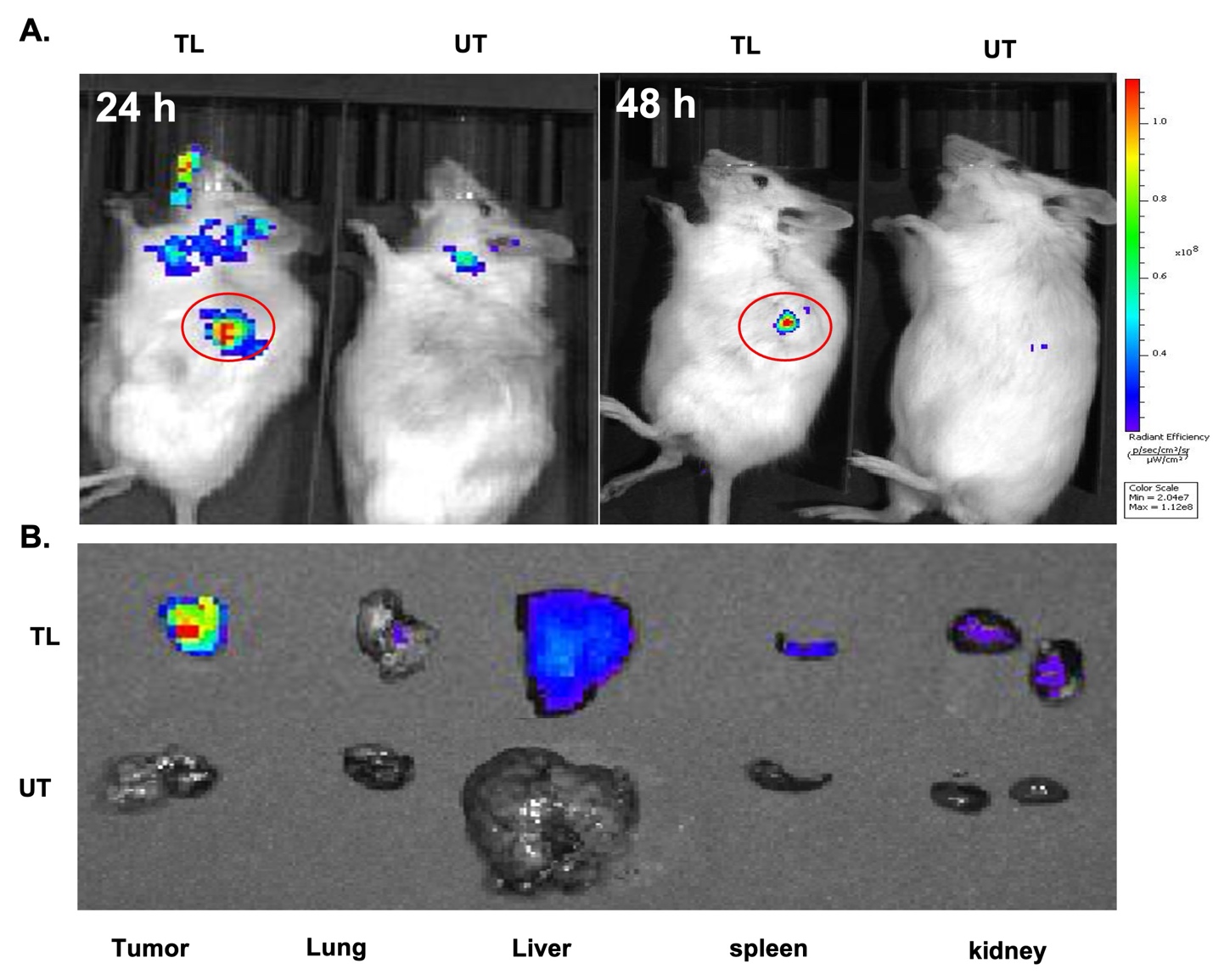


**Figure S3:** in vivo selective tumor uptake of targeted liposomal formulations: IVIS imaging showing tumor accumulation of Cy5.5-siRNA complexed targeted liposomal formulations 24 h and 48 h of after i.p. administration into mice bearing orthotopic PANC-1 tumor. Cy 5.5 -siRNA complexed targeted liposomal formulations show significantly higher tumor accumulation.


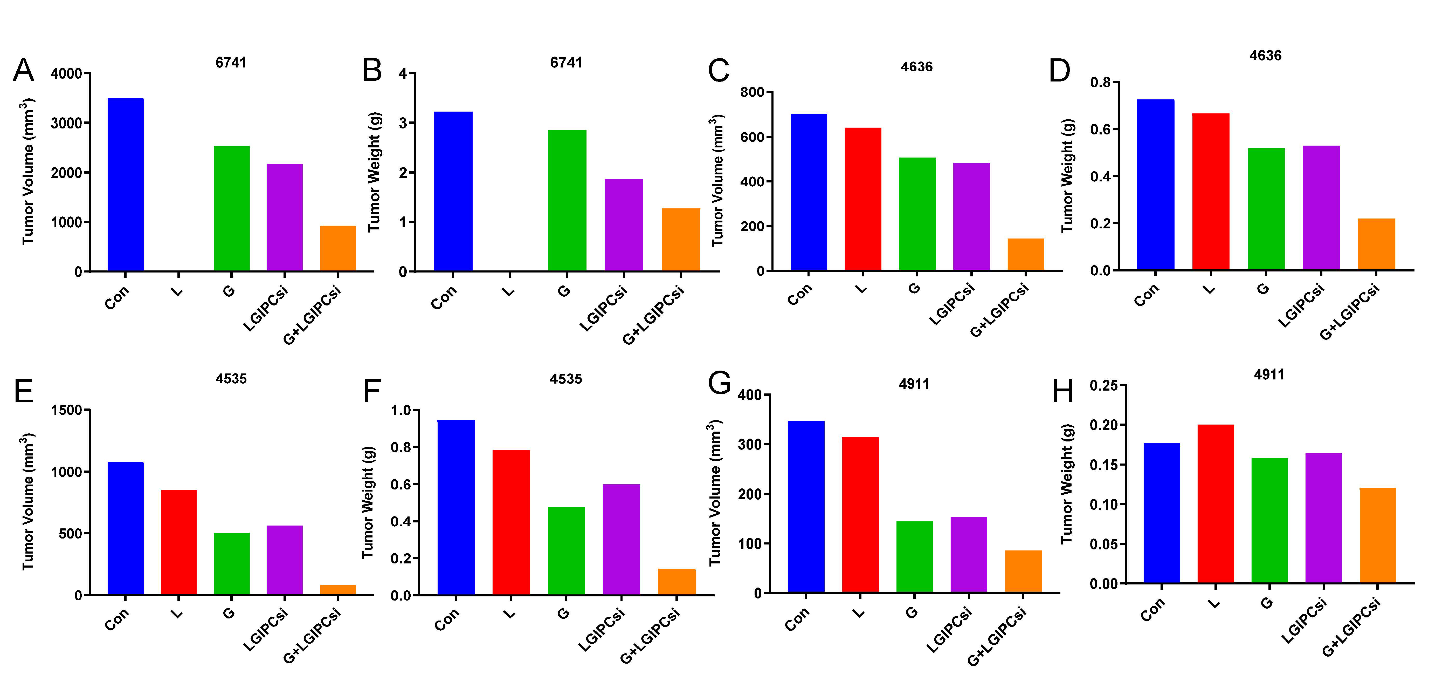


**Figure S4:** Anti-tumor effects of combination of downregulating of GIPC-1 and Gemcitabine treatments in in single mice experiment and validation in pancreatic xenograft mouse model: Mice bearing 6741, 4636, 4535 and 4911 orthotopic pancreatic tumors were treated with five different combinations Control (n = 1) Empty liposome(D1XP), Gemcitabine (10 mg/kg), GIPC-1 siRNA( 0.5mg/kg), and both Gemcitabine (10 mg/kg), GIPC-1 siRNA( 0.5 mg/kg) for over 2x/3 weeks. Untreated group used as control. A and B Tumor volume and Tumor weight of 6741, C and D tumor volume and tumor weight of 4636, E and F tumor volume and tumor weight of 4535 tumors and G and H tumor volume and tumor weight of 4911 tumors. The antitumor efficacy of gemcitabine combined with GIPC-1 siRNA treated mice significantly inhibited tumor growth comparing with only gemcitabine treated or only GIPC siRNA treated or control mice.
